# Regional, socioeconomic, and dietary factors influencing B-vitamins in human milk of urban Chinese lactating women at different lactation stages

**DOI:** 10.1186/s40795-017-0139-1

**Published:** 2017-03-07

**Authors:** Yong Xue, Karine Meisser Redeuil, Esther Campos Giménez, Gerard Vinyes-Pares, Ai Zhao, Tingchao He, Xiaoguang Yang, Yingdong Zheng, Yumei Zhang, Peiyu Wang, Sagar K. Thakkar

**Affiliations:** 1 Department of Nutrition & Food Hygiene, School of Public Health, Peking University Health Science Center, 38 Xueyuan Road, Haidian District, Beijing, 100191 People’s Republic of China; 2 CAS key Laboratory of Pathogenic Microbiology and Immunology, Institute of Microbiology, Chinese Academy of Science, Chaoyang District, Beijing, 100101 People’s Republic of China; 3 Nestlé Research Center, Lausanne, Vers-chez-les-Blanc, 1000 Lausanne, Switzerland; 4 Nestlé Research Center, Building E-F, No.5 Dijin Road, Haidian District, Beijing, 100095 People’s Republic of China; 5 Department of Social Medicine & Health Education, School of Public Health, Peking University Health Science Center, Haidian District, Beijing, 100191 People’s Republic of China; 6 National Institute for Nutrition and Health, Chinese Centre for Disease Control and Prevention, Chaoyang District, Beijing, 100050 People’s Republic of China; 7 Department of Epidemiology & Biostatistics, School of Public Health, Peking University Health Science Center, Haidian District, Beijing, 100191 People’s Republic of China

**Keywords:** Human milk, B-vitamins, Cross-sectional study, High performance liquid chromatography-tandem mass spectrometry, Microbiological assays

## Abstract

**Background:**

Adequate B-vitamins concentrations in human milk are considered to be a prerequisite for healthy development of infants in early life. This study aims to determine the concentrations of B-vitamins in human milk from Chinese women and the relationships between their concentrations and different geographical origin, lactation stages, socioeconomic characteristics, and dietary intake.

**Methods:**

Human milk was obtained from 443 healthy lactating women from Beijing (*n* = 150), Suzhou (*n* = 146), and Guangzhou (*n* = 147) cities. Thiamine, riboflavin, vitamin B_3_ (nicotinamide and nicotinic acid), and vitamin B_6_ (pyridoxal, pyridoxine, and pyridoxamine) in human milk were analyzed by high performance liquid chromatography-tandem mass spectrometry. Pantothenic acid, biotin, and folates in human milk were analyzed by microbiological assay. The information from one 24-h dietary recall and socioeconomic characteristics were collected by interview and structured questionnaire, respectively.

**Results:**

B-vitamins concentrations in human milk varied greatly among individuals. The median concentrations of B-vitamins of *postpartum* 5–11 d, 12–30 d, 31–60 d, 61–120 d, and 121–240 d were respectively as follows: thiamine 3.13, 5.07, 4.28, 5.65, 6.28 (μg/100 g); riboflavin 20.8, 20.2, 11.9, 13.6, 15.6 (μg/100 g); vitamin B_3_ 194.0, 300.0, 261.0, 212.5, 218.0 (μg/100 g); pantothenic acid 236.5, 291.0, 254.0, 179.0, 189.0 (μg/100 g); vitamin B_6_ 6.34, 7.58, 8.60, 9.34, 10.20 (μg/100 g); biotin 0.462, 0.834, 0.606, 0.523, 0.464 (μg/100 g); folates 0.730, 2.390, 2.440, 2.420, 2.330 (μg/100 g). The levels of B-vitamins presented regional differences and varied significantly among different lactation stages. The inversely associations of thiamine, vitamin B_6_, and folates with maternal BMI were found in multivariate analyses (*p* < 0.05), as well as higher pantothenic acid, folates, and biotin concentrations in lactating women with supplement intake when compared with those without (*p* < 0.05). Riboflavin concentrations associated with regular exercise was found in multivariate analyses (*p* < 0.05).

**Conclusions:**

The present study indicated regional and socioeconomic factors, lactation stage, and supplement intake may influence B-vitamins concentrations of human milk in healthy Chinese mothers. Further studies on accurate and complete analysis of all vitamin forms are crucial for giving a more comprehensive understanding of vitamin status in human milk.

**Trial registration:**

ClinicalTrials.gov, NCT01971671. Registered 13 October 2013.

**Electronic supplementary material:**

The online version of this article (doi:10.1186/s40795-017-0139-1) contains supplementary material, which is available to authorized users.

## Background

According to the global strategy for Infant and Young Child Feeding from World Health Organization, exclusive breastfeeding is recommended during the first 6 months of life to ensure an optimal growth, development, and health of the infant [1, 2]. Thereafter, complementary foods should be introduced while breastfeeding continues having a critical role in providing adequate nutrition through infancy [3]. Hence, in order to provide a primary knowledge basis for estimates of infant vitamin requirements and recommended levels of intakes, it is necessary to accurately measure the concentration of nutrients in human milk [4].

B-vitamins including thiamine, riboflavin, niacin, pantothenic acid, vitamin B_6_, biotin, and folates are essential nutrients for maternal health during pregnancy and lactation and particularly important for the growth of infant [5–7]. Their deficiency can affect pregnancy outcome [5, 8], as well as dermal [9, 10], blood [8], nervous [11], and digestive system [5, 6], even mortality of infant [8, 9]. Numerous researches [5, 6, 8, 11–15] showed that the contents of B-vitamins in human milk were significantly affected by maternal status and dietary intake. Moreover, maternal deficiency can rapidly result in very low concentrations of B-vitamins in human milk [5], which has been shown to improve with dietary supplements [8, 16–20].

Most of the researches on B-vitamins have been limited to Caucasian [11, 15–17, 19–23], East Asian (i.e. Japanese and Korean) [4, 24, 25], and Africa populations [26–28] with little data from Southeast Asian [18] and American populations [29, 30]. In China, one study [12] on human milk from Inner Mongolia reported some B-vitamins, but the significance of its result was limited because of its small sample size (Colostrum: *n* = 7; Transitional milk: *n* = 7; Mature milk: *n* = 66). Another multiregional study [14] on human milk with larger sample size determined B-vitamins in Chinese lactating women, and analyzed their differences among different lactation stages and different areas. However, the associations between B-vitamins in human milk and maternal socioeconomic and dietary factors were not explored, which limits the understanding of human milk composition.

Several factors have been suggested to impact the secretion of B-vitamins in human milk, such as nutritional status of the mothers during pregnancy [31], supplementation [8, 16–20], diurnal variation [32], and preterm status of the infants [33]. In addition, maternal age [34], body weight index (BMI), education, income, and regular exercise [35] may also modify B-vitamins status in human milk. Additionally, in China, the swift of the traditional dietary pattern due to social, economic, and health transition in the past few decades [36], might have impacted the contents of human milk.

The aims of this study were to determine the composition of B-vitamins including thiamine, riboflavin, vitamin B_3_ (nicotinamide and nicotinic acid), pantothenic acid, vitamin B_6_ (pyridoxine, pyridoxamine, and pyridoxal), biotin, and folates in breast milk of lactating mothers at 0–8 m *postpartum* from urban areas of China; to evaluate their interregional differences and to explore associations with nutrient intake. In addition, the associations with maternal and obstetric characteristics were also investigated. This study is part of the larger initiative Maternal Infant Nutrition Growth (MING) study.

## Methods

### Study design

**Fig. 1:**
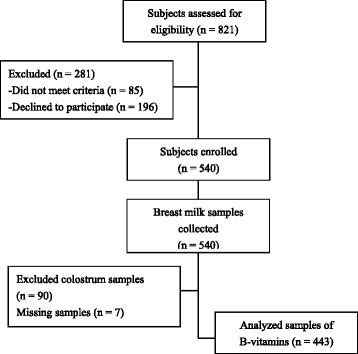
Study flow chart subjects enrolled

As one part of the MING study which was designed to research the dietary and nutritional status of pregnant women, lactating mothers and young children aged 0–3 years living in urban areas of China, this study was focused on B-vitamins status in breast milk. A multi-stage sampling was applied in this cross-sectional study: firstly, three cities (Beijing is located in Northern China; Suzhou: Eastern China; Guangzhou: Southern China) were chosen according to the geographical location and status of economic development; secondly, one grade three and first-class hospital and one maternal & child hospital were selected from each city considering the scale of hospital and the feasibility of investigation; thirdly, 443 apparently healthy, well-nourished lactating women aged 18–42 years were recruited from registration records in aforementioned hospitals between October 2011 and February 2012, including 89 mothers of 5–11 d postpartum, 87 of 12–30 d postpartum, 89 of 31–60 d postpartum, 90 of 61–120 d postpartum, and 88 of 121–240 d postpartum. Study subjects were selected using the following criteria: 1) aged 18–45 years, 2) give birth to a single gestation, 3) having full-term and healthy child. Lactating women with self-reported diabetes, hypertension, cardiac diseases, and/or acute communicable diseases, or women who took hormone in recent 3 months, were ineligible for the study, as were those with postpartum depression or insufficient skills to understand study questionnaire. Figure 1 displays the recruitment flowchart from eligibility to sample analysis.

### Data collection

Through face-to-face interviews, the structured questionnaire was used to collect socio-economic characteristics and lifestyle aspects of lactating women including maternal age, educational level, drinking, smoking, and family’s per capita income. The information of regular exercise defined as exercising more than once a week and lasting more than 30 min every time. Delivery mode and number of gestational weeks at delivery were also recorded. Additionally, anthropometric parameters such as height and weight were measured by the dedicated researchers. BMI (kg/m^2^) of mothers was calculated using height and current weight, and categorized as underweight (BMI < 18.5 kg/m^2^), normal weight (18.5–24.9 kg/m^2^), overweight (25.0–29.9 kg/m^2^), or obesity (≥30 kg/m^2^). In order to exclude those lactating mothers with hypertension or diabetes mellitus, the information of blood pressure and blood glucose were also collected from hospital records or maternal health records. Besides, gender information and date of birth of the baby was collected by telephone interview after the data collection since the data was not included in the initial questionnaires.

### Milk collection

All human milk samples were collected in a dim light room in hospitals without direct sunlight exposure. After the women had breakfast, the samples were collected at the second feeding in the morning to avoid circadian influence on the outcomes (9–11 am). Before collection of milk, breasts of the subjects (which were emptied by mother herself during 6 am to 7 am) were warmed about 5–10 min by hot towel, then one full breast was emptied by trained investigators using an electric breast pump (Horigen HNR/X-2108ZB, Xinhe Electrical Apparatuses Co., Ltd., Guangzhou, China) and the milk was collected into a feeding bottle. After gently up-down shaking for ~10 times, a sample was taken (15 mL for 5–11 d *postpartum*, 40 mL for 12–30 d *postpartum*, 31–60 d *postpartum*, 61–120 d *postpartum*, and 121–240 d *postpartum*, respectively) and then shipped to the lab using portable incubator filled with ice bags within 1 h. The rest of the milk was returned to the mother for feeding to the infant. The samples were divided with 1 mL freezing tubes under the dim light on ice, labeled with subject number, and then frozen at -80 °C until analysis.

### Analysis of B-vitamins in human milk

All of the milk samples were analyzed by Eurofins Technology Service (Suzhou) Co.Ltd in China with various analytical methods as mentioned below. Only vitamers listed in brackets were quantified in this study.

*Methodology for quantifying Vitamin B*_*1*_ *(thiamine), Vitamin B*_*2*_ *(riboflavin), Vitamin B*_*3*_ *(nicotinamide and nicotinic acid), and Vitamin B*_*6*_ *(pyridoxal, pyridoxine and pyridoxamine)*: acidic hydrolysis followed by high performance liquid chromatography-tandem mass spectrometry (HPLC-MS/MS) based quantification.

One gram of sample was weighted in 15 mL centrifuge tube and hydrolyzed with 0.5 mL of hydrochloric acid (1 M) in autoclave for 30 min at 120 °C. Samples were cooled down and pH was then adjusted to 4.5 ± 0.5 by addition of HCl or NaOH. 0.01 g of taka-diastase was added to the samples, placed in water bath at 45 °C for 3 h. Sample volume was then adjusted to 10 mL with water and filtrated through 0.45 μm filter. 1 mL of sample extract was taken and isotopic labeled internal standards (thiamine-[^13^C_4_], riboflavin-[^13^C_4_, ^15^N_2_], nicotinic acid-d_4_, nicotinamide-d_4_, pyridoxine-d_2_, pyridoxal-[^2^H_3_] and pyridoxamine-[^2^H_3_] at a concentration of 200 μg/L) were added. Sample analysis was performed on a HPLC-MS/MS system. Liquid chromatography (LC) was performed using an Agilent 1100 (Agilent Technologies, Waldbronn, Germany) LC system. Chromatographic separation was carried out with a Waters Acquity HSS T3 column (2.1 × 100 mm i.d., 1.8 μm). Column temperature was set to 30 °C and the autosampler remained stable at 15 °C. Ammonium formate aqueous solution (2.5 mM, solvent A) and acetonitrile (solvent B) served as mobile phase at a flow rate of 0.6 mL/min. The gradient process was: 0 min, 75% A; 1 min, 75% A; 1.1–10 min, 50% A; 10.1–15 min, 50% A; 15.1–16.5 min, 75% A; 16.6–0 min, 75% A. An API 4000 triple quadrupole mass spectrometer (Applied Biosystems, Foster City, CA, USA) was used for detection of the chromatographic separation. Nestle Internal reference material (bovine milk-based infant formula) was used for method validation and quality control (QC) sample as well, reference values were established by Nestle Proficiency Test participated by Nestle laboratories and third party laboratories. Coefficient of variation of repeatability is ranged from 3.7 to 13% and Coefficient of variation of intermediate reproducibility is ranged from 5.4 to 12%. The internal reference samples were regularly included and analyzed in duplicate during analytical runs. The limit of detection (LOD) for thiamine, riboflavin, vitamin B_3_ and vitamin B_6_ were 0.65, 1, 1, and 0.65 (μg/100 g), respectively. Recovery rates of vitamin B_1_, vitamin B_2_, vitamin B_3_, and vitamin B_6_ were ranged from 92 to 107%. Cross-talking in scheduled multiple reaction monitoring (MRM) was not observed between internal standards and analytes. The result of Vitamin B_3_ is the sum of nicotinamide and nicotinic acid, and it is expressed as nicotinic acid (niacin). As the mol masses are nearly identical both masses are only summed up. The result for Vitamin B6 is reported as pyridoxine. Thus pyridoxal hydrochloride (correction factor 0.831) and pyridoxamine dihydrochloride (correction factor 0.702) are converted to pyridoxine.

*Methodology for quantifying pantothenic acid, biotin, and folates:* solvent extraction followed by microbiological assay based quantification.

One gram of sample was weighed and diluted with deionized water (40 mL). Sample extraction was then performed in a water bath at 95 °C for 30 min. After centrifugation (8000 g, 5 min), sample extracts could be further diluted with sterile water provided from the test kit if needed. Sample extracts were then pipetted on the commercially available microtiter plate VitaFast^®^ (R-Biopharm Analysis System Trading Ltd, Beijing, China). After having applied all kit instructions including microorganism incubation at 37 °C in the dark for 20–24 h (pantothenic acid) or 44–48 h (biotin and folates), the turbidity of each vial was measured with a microtiter plate reader at 610–630 nm (alternatively at 540–550 nm), for the calculation of pantothenic acid, biotin, and folates concentration. The LOD values for pantothenic acid, biotin, and folates were 0.074, 0.080, and 0.160 (μg/100 g), respectively. The measurements of QC samples described above (internal reference samples) at predetermined intervals were regularly performed to obtain QC curve for the demonstration of method’s reliability. Recovery rates of pantothenic acid, biotin, and folates were ranged from 93 to 112%.

### Dietary measurement

Experienced and trained researchers conducting dietary interviews obtained the information of dietary recall during the previous 24-h from participants by face-to-face interview when milk samples were collected. All of the foods intakes were coded and B-vitamins (including thiamine, riboflavin, and niacin intakes) were analyzed using a database according to Chinese Food Composition (CFC) tables 2004 & 2009 consist of 1773 food items [37, 38]. Considering the fact that vitamin B-6, biotin, and folic acid were detected in very limited food items in CFC, the Japan Food Composition (JFC) tables (2014) [39] was used to estimate intakes of them in lactating women. If the participants had consumed dietary supplements, they were asked to supply the basic supplement information, including the brand name, manufacturer, and actual consumption (daily dosage).

### Statistical analysis

Socioeconomic characteristics of lactating women were described as count (percentage) for categorical variables and median value with interquartile range for continuous variables without normal distribution. Chi-squared tests (categorical variables) and Kruskal-Wallis tests (continuous variables) were used to compare participants’ characteristic according to stages of lactating period. Median (interquartile range) and mean ± standard deviation were calculated for each of B-vitamin according to research cities (Beijing, Suzhou, and Guangzhou cities) and stages of lactation (5–11 d *postpartum*, 12–30 d *postpartum*, 31–60 d *postpartum*, 61–120 d *postpartum*, and 121–240 d *postpartum*). Before the progress of analysis about B-vitamins, Shapiro-Wilk test was employed to determine whether B-vitamins in human milk had a normal distribution or not. Because of non-normal distribution in human milk vitamins, naturally logarithmic transformations were applied when doing analysis of variance (ANOVA) according to research cities and lactating stages. Multivariate linear regression analysis was used to describe the relationship between B-vitamins concentrations in breast milk (dependent variables) and socioeconomic characteristics (independent variable) of lactation women. A stepwise forward selection process was used in which the independent variables and confounders (maternal age, present BMI, education, family income, delivery mode, regular exercise, supplement intake, duration of breastfeeding, and city) were added to the models according to their significance (*p* value). Homogeneity and bias was showed in the analyses of the residuals of the final models. The associations between B-vitamins concentrations and diet characteristics such as dietary B-vitamins intakes were evaluated by partial-correlations adjusted for research cities and lactation stages. All statistical analyses were performed by using the SPSS software (Ver. 20.0) (SPSS Inc. Chicago, IL, USA), and the level of significance was set at *p* < 0.05 based on a two-sided calculation.

## Results

The socioeconomic characteristics of the lactating women are summarized in Table 1. The mean age of the lactating women was 27.4 ± 4.0 years. The majority of lactating women had completed high school and had a monthly household income representative of urban China. Although the majority of women had normal BMI at present, up to 45% lactating women had a cesarean delivery. Only 11.1% lactating women were consuming dietary supplements. According to the stage of lactation, significant differences were found in mode of delivery and educational level. These differences were taken into consideration for the analyses of concentration over lactation and the statistical model were adjusted for these potential confounding factors.

**Table 1:** Socioeconomic characteristics of lactating mothers with different stages of lactating period

	5–11 d (*n* = 89)	12–30 d (*n* = 87)	31–60 days (*n* = 89)	61–120 d (*n* = 90)	121–240 d (*n* = 88)	*P*-value
Age, years ^1^						0.120
< 25	26 (29.2)	27 (31.0)	18 (20.2)	26 (28.9)	34 (38.6)	
25–30	42 (47.2)	38 (43.7)	44 (49.4)	50 (55.6)	39 (44.3)	
> 30	21 (23.6)	22 (25.3)	27 (30.3)	14 (15.6)	15 (17.0)	
Offspring gender ^1^						0.729
Male	50 (56.2)	46 (52.9)	47 (52.8)	54 (60.0)	42 (47.7)	
Female	39 (43.8)	37 (42.5)	39 (43.8)	36 (40.0)	43 (48.9)	
Education ^1^						<0.001 ^*^
Middle school or blow	11 (12.4) ^a^	15 (17.2) ^a, b^	26 (29.2) ^b^	22 (24.4) ^a, b^	38 (43.2) ^c^	
High school	31 (39.1)	34 (39.1)	21 (23.6)	25 (27.8)	23 (26.1)	
College or above	45 (42.5)	37 (42.5)	42 (47.2)	41 (45.6)	26 (29.5)	
Family’s per capita income, Yuan/month ^1^						0.165
< 2000	20 (22.5)	17 (19.5)	23 (25.8)	26 (28.9)	31 (35.2)	
2000–4000	36 (40.4)	43 (49.4)	41 (46.1)	40 (44.4)	39 (44.3)	
> 4000	30 (33.7)	21 (24.1)	23 (25.8)	22 (24.4)	18 (20.5)	
Unclear	3 (3.4)	6 (6.9)	2 (2.2)	2 (2.2)	0 (0.0)	
Delivery mode ^1^						0.038 ^*^
Vaginal	50 (56.2) ^a, b^	45 (51.7) ^a^	37 (41.6) ^b^	55 (61.1) ^a^	54 (61.4) ^a^	
Cesarean	37 (41.6)	42 (48.3)	52 (58.4)	35 (38.9)	33 (37.5)	
Present BMI ^1^						0.109
Underweight	5 (5.6)	2 (2.3)	2 (2.2)	4 (4.4)	7 (8.0)	
Normal	54 (60.7)	58 (66.7)	56 (62.9)	69 (76.7)	64 (72.7)	
Overweight	26 (29.2)	26 (29.9)	26 (29.2)	16 (17.8)	16 (18.2)	
Obesity	3 (3.4)	1 (1.1)	5 (5.6)	1 (1.1)	1 (1.1)	
Dietary supplements intake ^1^						0.810
Yes	10 (11.2)	12 (13.8)	11 (12.4)	8 (8.9)	8 (9.1)	
No	79 (88.8)	75 (86.2)	78 (87.6)	82 (91.1)	80 (90.9)	
Pregnancy duration, weeks ^2^	39 (39, 40)	39 (39, 40)	39 (38, 40)	39.5 (39, 40)	40 (39, 40)	0.268

Data were expressed as median (interquartile range) for continuous variables without normal distribution and count (percentage) for categorical variables

*BMI* body mass index, was calculated as body weight by height squared (kg/m^2^)

^1^ Compared by chi-square test

^2^ Compared by Kruskal-Wallis test

^*^ Indicates a significant difference among six stages of lactating period (*p* < 0.05)

^a, b, c^ Data with the different superscript letters in the same row differ significantly (*p* < 0.05); Difference between two subgroups using Chi-squared tests (categorical variables) and Mann-Whitney U test (continuous variables without normal distribution)

The selected methodologies in this study allowed us the quantification of thiamine as B_1_ marker; riboflavin as B_2_ marker, nicotinamide and nicotinic acid as B_3_ marker and the sum of pyridoxine, pyridoxamine and pyridoxal as B_6_ marker. Pantothenic acid concentration is presented as total pantothenic acid. Folates concentration is presented as free folates concentration. However, the HPLC-MS/MS method did not allow to also quantify thiamine monophosphate (TMP) (B_1_ vitamer), flavin adenine dinucleotide (FAD) and flavin mononucleotide (FMN) (B_2_ vitamer) and pyridoxal 5’-phosphate (PLP) (B_6_ vitamer) Therefore, these vitamin concentration are likely to be underestimated.

The concentrations of B-vitamins in different stages of lactation are shown in Table 2. Variations of B-vitamins concentrations according to stage of lactation were observed in this study. Overall, mean concentrations of thiamine, vitamin B_6_ and folates increased progressively with duration of lactation (*p* for trend < 0.001). In contrast, riboflavin and pantothenic acid concentrations fell over time (*p* for trend < 0.01). Unlike the other vitamins, the levels of vitamin B_3_ and biotin remained almost constant as lactation stage increased (*p* for trend > 0.05).

**Table 2:** B-vitamins concentrations of milk samples during different lactation stages ^1, 2^

B-group vitamin	5–11 d	12–30 d	31–60 d	61–120 d	121–240 d
	(*n* = 89)	(*n* = 87)	(*n* = 89)	(*n* = 90)	(*n* = 88)
Thiamine, μg/100 g					
n	44	73	82	89	86
Median (IQR)	3.13 (2.58, 4.89) ^a^	5.07 (3.11, 6.47) ^b^	4.28 (3.06, 6.61) ^b^	5.65 (3.78, 7.69) ^c^	6.28 (5.11, 8.03) ^d^
Mean ± SD	3.60 ± 1.29	5.01 ± 2.10	4.69 ± 1.85	5.75 ± 2.18	6.69 ± 2.17
Riboflavin, μg/100 g					
n	88	86	83	89	88
Median (IQR)	20.8 (13.2, 31.5) ^a^	20.2 (10.1, 27.4) ^b^	11.9 (7.1, 21.1) ^c^	13.6 (9.7, 20.1) ^c, d^	15.6 (12.3, 19.4) ^b, d^
Mean ± SD	25.4 ± 18.8	19.4 ± 9.9	15.3 ± 12.0	15.1 ± 7.6	16.4 ± 7.1
Vitamin B_3_^3^, μg/100 g					
n	89	87	89	90	88
Median (IQR)	194.0 (110.0, 320.5) ^a^	300.0 (248.0, 378.0) ^b^	261.0 (183.0, 323.5) ^c^	212.5 (168.8, 277.3) ^a, d^	218.0 (168.8, 328.8) ^c, d^
Mean ± SD	239.1 ± 156.3	337.1 ± 151.4	272.1 ± 118.4	227.8 ± 82.6	253.6 ± 118.9
Pantothenic acid, μg/100 g					
n	86	84	89	90	
Median (IQR)	236.5 (166.3, 324.3) ^a^	291.0 (229.5, 374.3) ^b^	254.0 (187.0, 346.5) ^a^	179.0 (154.5, 220.0) ^c^	189.0 (153.0, 251.5) ^c^
Mean ± SD	255.1 ± 117.9	304.0 ± 109.6	264.2 ± 94.6	204.2 ± 79.5	205.8 ± 63.2
Vitamin B_6_^4^, μg/100 g					
n	60	75	89	89	87
Median (IQR)	6.34 (3.83, 9.85) ^a^	7.58 (5.92, 9.86) ^a, b^	8.60 (6.32, 10.55) ^b^	9.34 (7.40, 12.00) ^c^	10.20 (8.15, 13.80) ^c^
Mean ± SD	8.63 ± 7.57	8.22 ± 4.10	8.94 ± 3.86	10.30 ± 4.91	10.90 ± 4.37
Biotin, μg/100 g					
n	78	84	88	90	87
Median (IQR)	0.462 (0.187, 0.856) ^a^	0.834 (0.550, 1.190) ^b^	0.606 (0.435, 0.876) ^c^	0.523 (0.366, 0.749) ^a, c^	0.464 (0.316, 0.648) ^a^
Mean ± SD	0.691 ± 0.795	0.967 ± 0.703	0.701 ± 0.424	0.617 ± 0.429	0.577 ± 0.627
Folates, μg/100 g					
n	88	87	89	90	88
Median (IQR)	0.730 (0.387, 1.245) ^a^	2.390 (1.340, 3.120) ^b^	2.440 (1.615, 3.440) ^b, c^	2.420 (1.653, 3.265) ^c^	2.330 (1.515, 3.875) ^c^
Mean ± SD	1.072 ± 0.945	2.421 ± 1.379	2.665 ± 1.366	2.759 ± 1.583	2.860 ± 1.694

*IQR* interquartile range; *SD* standard deviation

^1^ Data were presented as the median (IQR) and mean ± SD

^2^ Compared by One-Way analysis of variance (ANOVA) after the ln transformation followed by Fisher’s least significant difference (LSD) post hoc comparisons

^3^ Vitamin B_3_ = nicotinamide + nicotinic acid

^4^ Vitamin B_6_ = pyridoxine + 0.702pyridoxamine + 0.831pyridoxal

^a, b, c, d^ Data with the different superscript letters in the same row differ significantly (*p* < 0.05)

The concentrations of B-vitamins in human milk from Chinese mothers living in Beijing, Suzhou, and Guangzhou cities are shown in Table 3. In this study, wide range of variations in B-vitamins content was found. Interregional differences of concentrations were present in the majority of B-vitamins (*p* < 0.05) except for biotin in human milk. The concentrations of thiamine and riboflavin were significantly higher in human milk from Beijing city than those from Suzhou and Guangzhou cities (*p* < 0.001). The concentrations of vitamin B_3_ and vitamin B_6_ were significantly lower in human milk from Suzhou city than the other two cities (*p* < 0.05). The level of pantothenic acid was significantly higher in human milk from Suzhou city than those from Guangzhou (*p* < 0.05). Meanwhile, the folates content in human milk from Suzhou city was significantly higher than that from Beijing city (*p* < 0.001).

**Table 3:** B-vitamins concentrations of milk samples from lactating mothers in the three cities^1,2^

B-group vitamin	City1: Beijing	City2: Suzhou	City3: Guangzhou	Total
	(*n* = 150)	(*n* = 146)	(*n* = 147)	(*n* = 443)
Thiamine, μg/100 g				
n	134	108	132	374
Median (IQR)	6.61 (5.21, 7.89) ^a^	4.25 (2.74, 6.38) ^b^	4.40 (3.49, 5.60) ^b^	5.17 (3.54, 6.81)
Mean ± SD	6.41 ± 1.98	4.90 ± 2.67	4.60 ± 1.52	5.34 ± 2.22
Riboflavin, μg/100 g				
n	150	139	145	434
Median (IQR)	20.1 (14.5, 25.1) ^a^	12.5 (6.6, 19.3) ^b^	14.8 (9.5, 23.1) ^c^	16.2 (10.0, 23.5)
Mean ± SD	20.7 ± 8.06	16.7 ± 17.1	17.4 ± 10.4	18.3 ± 12.4
Vitamin B_3_^3^, μg/100 g				
n	150	146	147	443
Median (IQR)	264.0 (185.8, 349.0) ^a^	212.0 (151.5, 291.8) ^b^	244.0 (179.0, 343.0) ^a^	240.0 (171.0, 322.0)
Mean ± SD	274.0 ± 124.4	226.4 ± 94.3	295.9 ± 162.9	265.6 ± 133.2
Pantothenic acid, μg/100 g				
n	148	146	143	437
Median (IQR)	230.0 (184.5, 299.5) ^a,b^	225.0 (174.5, 339.8) ^a^	199.0 (155.0, 282.0) ^b^	223.0 (166.5, 302.0)
Mean ± SD	245.7 ± 78.7	261.9 ± 121.9	230.1 ± 97.8	245.9 ± 101.6
Vitamin B_6_^4^, μg/100 g				
n	139	132	129	400
Median (IQR)	8.37 (6.52, 10.90) ^a^	7.27 (4.64, 10.2) ^b^	9.46 (8.22, 13.30) ^c^	8.63 (6.43, 11.00)
Mean ± SD	9.06 ± 3.37	8.57 ± 6.35	10.89 ± 4.74	9.49 ± 5.04
Biotin, μg/100 g				
n	144	142	141	427
Median (IQR)	0.585 (0.385, 0.878) ^a^	0.554 (0.325, 0.875) ^a^	0.554 (0.314, 0.846) ^a^	0.568 (0.336, 0.858)
Mean ± SD	0.749 ± 0.674	0.691 ± 0.585	0.685 ± 0.596	0.709 ± 0.619
Folates, μg/100 g				
n	149	146	147	442
Median (IQR)	1.790 (1.125, 2.710) ^a^	2.400 (1.190, 3.428) ^b^	2.220 (1.230, 3.420) ^a,b^	2.140 (1.190, 3.143)
Mean ± SD	1.998 ± 1.226	2.635 ± 1.766	2.446 ± 1.576	2.358 ± 1.557

*IQR* interquartile range; *SD* standard deviation

^1^ Data were presented as the median (IQR) and mean ± SD

^2^ Compared by One-Way analysis of variance (ANOVA) after the ln transformation followed by Fisher’s least significant difference (LSD) post hoc comparisons

^3^ Vitamin B_3_ = nicotinamide + nicotinic acid

^4^ Vitamin B_6_ = pyridoxine + 0.702pyridoxamine + 0.831pyridoxal

^a,b,c^ Data with the different superscript letters in the same row differ significantly (*p* < 0.05)

No significant correlations were observed between B-vitamins and maternal age, educational level, family income, and delivery mode in single factor analysis (*p* > 0.05). The adjusted associations between B-vitamin concentrations in human milk and the socio-economic characteristics of lactating women are summarized in Table 4 and Additional file 1: Table S1. In multivariate analyses, compared with lactating women with normal BMI, those overweight women had significantly lower thiamine concentrations in human milk after adjustment for potentially confounding factors (*p* < 0.05). Additionally, compared with lactating women with normal BMI, lower vitamin B_6_ in those overweight and obesity women and higher folates in those underweight women were found in this study (*p* < 0.05, *p* < 0.05, and *p* < 0.01, respectively). After adjustment for potentially confounding factors, significantly higher pantothenic acid, biotin, and folates were observed in lactating women with dietary supplement intake when compared with the other women (*p* < 0.01, *p* < 0.001, and *p* < 0.05, respectively). Meanwhile, the concentrations of riboflavin in human milk from the lactating women with regular exercise were significantly higher than those women without (*p* < 0.05).

**Table 4:** Multivariate linear regression models considering thiamine, pantothenic acid, vitamin B_6_ and folates concentrations in human milk after the ln transformation as the dependent variables and the other variables studied as independent variables

	Thiamine		Pantothenic acid		Vitamin B_6_		Folates	
	β (95% CI) ^a^	*P*-value	β (95% CI) ^a^	*P*-value	β (95% CI) ^a^	*P*-value	β (95% CI) ^a^	*P*-value
BMI, kg/m^2^								
< 18.5	0.168 (−0.106, 0.442)	0.228			0.098 (−0.251, 0.446)	0.582	0.454 (0.166, 0.743)	0.002
18.5–24.9	Ref				Ref		Ref	
25–29.9	−0.150 (−0.284, −0.015)	0.029			−0.203 (−0.374, −0.032)	0.020	−0.138 (−0.279, 0.004)	0.056
≥ 30	−0.298 (−0.667, 0.071)	0.114			−0.583 (−1.052, −0.114)	0.015	−0.044 (−0.432, 0.343)	0.822
Supplement intake								
Yes			0.188 (0.061, 0.315)	0.004			0.206 (0.015, 0.396)	0.034
No			Ref				Ref	

Adjusted R^2^ for thiamine = 0.463, *p* < 0.001; adjusted R^2^ for pantothenic acid = 0.106, *p* < 0.001; adjusted R^2^ for vitamin B_6_ = 0.244, *p* < 0.001; adjusted R^2^ for folates = 0.350, *p* < 0.001

*BMI* body mass index; *CI* confidence interval; *Ref* reference

^a^ Adjusted for cities (Beijing, Suzhou, and Guangzhou cities) and lactation stages (*postpartum* 5–11 d, *postpartum* 12–30 d, *postpartum* 31–60 d, *postpartum* 61–120 d, and *postpartum* 121–240 d)

In partial-analyses, after adjustment for investigated cities and lactation stages, no significant correlations were observed between vitamins in human milk and the corresponding vitamins in dietary intakes (*r* = 0.015, *p* = 0.757 for thiamine; *r* = 0.046 and *p* = 0.353 for riboflavin, *r* = 0.034 and *p* = 0.489 for vitamin B_3_, *r* = 0.055 and *p* = 0.269 for vitamin B_6_, *r* = 0.082 and *p* = 0.101 for biotin, and *r* = −0.056 and *p* = 0.263 for folates, respectively).

## Discussion

When compared with previously reported results of investigations into human milk of Chinese lactating women, the same vitamers were analyzed and our results were generally comparable [12–14]. Though significant differences in riboflavin and vitamin B_3_ concentrations were found in this study when compared with other previous studies in China [13, 14], our results were similar to those in human milk from Inner Mongolia in China [12]. Generally, such results might be due to the differences in the milk sampling collection, loss during storage and pretreatment, and analytical methods [14], as well as dietary intake of study population. The methods for analysis of each B-vitamins including commonly microbiological, radioisotope dilution or more recently chromatographic, coupled with Ultra violet (UV), fluorometric and mass spectrometry (MS) detection [5]. HPLC-MS/MS methods in present study only analyze a specific form of Vitamin B_1_ (thiamine), Vitamin B_2_ (riboflavin), Vitamin B_3_ (nicotinamide and nicotinic acid), and Vitamin B_6_ (pyridoxal, pyridoxine and pyridoxamine) so that the contents of these vitamins analyzed by HPLC-MS/MS methods were much lower than the microbiological assays.

Ren et al. [14] reported that thiamine and vitamin B_6_ (pyridoxal, pyridoxine and pyridoxamine) concentrations increased with lactation stage increasing, which were consistent with our results. The finding from Ford et al. [40] and Sakurai et al. [4] showed total thiamin and total vitamin B_6_ increased progressively with duration of lactation, our results of only part of the total vitamin contents within these trends. However, riboflavin and pantothenic acid conce**n**trations decreased as the lactation stage increasing in the present study, which are contradictory with the previous studies [4, 40]. The results from Ren et al. [14] showed that riboflavin and pantothenic acid concentrations in milk from Chinese mothers reached a peak in transitional milk (*postpartum* 8–14 d) and then decreased with lactation stage increasing, which are consistent with our results. With lactation stage increasing, folic acid concentrations increased, which are similar to the trend of Ford et al. [40]. The research of Sakurai et al. [4] suggested that niacin (nicotinamide) and biotin levels remained nearly unchanged as lactation stage increased, which is consistent with our results. Generally, the main sources of water-soluble vitamins in milk are maternal plasma and dietary intake considering the inability of the mammary gland to synthetize such compounds, so the variations of those vitamins are mainly related to dietary intake and to nutritional regulation of the body [4].

Remarkably, dietary intakes and supplementations may play core roles in the levels of B-vitamins in human milk [11–14, 16–19]. Because of historical and cultural factors, cuisines in Beijing (located in north of China, inland area), Suzhou (located in east of China, lake area), Guangzhou (located in south of China, coastal area) are very different from each other [41], which may affect water-soluble vitamins intakes and result in significant differences of B-vitamins concentrations in human milk. Thiamine is largely present in cereals, particularly whole-grain cereals. In this study, the highest intake of cereals was found in Beijing city (309.5 g/day) when compared with those in Suzhou and Guangzhou cities (281.0 and 268.2 g/day). In addition, lactating women in Suzhou and Guangzhou main cereal source was refined rice (low in vitamin B_1_), Beijing had higher proportion of other cereals such as wheat and millet. This may explain the higher concentration of Thiamine in Beijing. Furthermore it is well known Riboflavin is widespread throughout many kinds of foods, especially from animal sources. Higher levels of riboflavin in human milk from Beijing city may be due to higher milk and eggs intake than those from Suzhou and Guangzhou cities. (219.8, 162.1, and 102.3 ml/day for milk intake, respectively; 68.8, 62.9, and 44.5 g/day for eggs intake, respectively). According to the results from 24 h dietary recall, highest intakes of folates were found in lactating women from Suzhou compared to others (294.9 vs 277.2 and 233.3 μg dietary folate equivalent for folic acid/day), and it was expected that folic acid were higher in human milk from Suzhou city. Actually, regional differences of water-soluble vitamins in human milk including between rural and urban area [13, 14], between coastal and inland area [14], and among difference countries [42] had been widely reported in previous studies.

According to the previous studies [43, 44], maternal nutrition practices/status related to vitamin concentrations in human milk is influenced by socio-economic status. However, little literature has focused on the relationships between the levels of water-soluble vitamins in human milk and the socio-economic characteristics of lactating women. In this study, significant correlations between levels of B-vitamins in human milk and maternal age, education, and family income were not found. These data suggest that even in the face of poor socioeconomic status, B-vitamins in milk maintained adequate levels when compared with populations in better socioeconomic status. In contrast, negative correlations between current BMI and some levels of B-vitamins in milk including thiamine, vitamin B_6_, and folates, were found in the present study. High postpartum weight retention is a strong independent risk factor for several chronic diseases such as obesity, cardiovascular disease, and type 2 diabetes [45], thus lactating women with postpartum overweight and obesity are more subject to control body weight. The inverse associations between maternal BMI and B-vitamins in milk may be due to postpartum diet control. Our results implied that regular exercise during lactation did not have negative impact on the B-vitamin concentrations, and were similar with the interview study from Lovelady et al. [35]. Furthermore, the highest riboflavin concentrations in milk in the present study were found from lactating women with regular exercise when compared with those women without. One possible explanation is that lactating women with regular exercise have stronger health consciousness and pay more attention on healthy diet which results in adequate food rich in vitamins intake. Studies [11, 12, 14, 15, 20, 46] have showed that the status of maternal nutrient supplements can influence the nutrient level of human milk, especially with regard to pantothenic acid, vitamin B_6_, biotin, and folates concentrations. Indeed, in this study, the levels of pantothenic acid, biotin, and folates found in human milk from women using vitamin supplement were markedly higher than those without, and these findings agreed with previous studies [16–20] on the fact that diets supplemented with B-vitamins had been associated with increase in concentrations.

Mother’s B-vitamins intake has been found to clearly correlate with their concentrations in human milk [11, 16–19]. West et al. [47] explored the influence of the level of vitamin B_6_ intake on the content of the vitamin in milk from 19 healthy subjects, and found significant correlation (*r* = 0.51, *p* < 0.01) between them. Similarly, Johnston et al. [15] found a significant positive correlation (*r* = 0.65, *p* < 0.01) between pantothenic acid in the diet of the mother the day preceding milk collection and the pantothenic acid content of the milk. Results of this study could not confirm this due to likely a limitation of the dietary intake assessment method [48] which only allowed for correlation of short-term dietary intake exposure with human milk composition. Nevertheless, this negative finding clearly suggest that to evaluate impact of dietary intake on human milk composition longer-term dietary intake assessments would be required.

Although state-of-the-art and/or reference analytical methodologies were applied, caution should be taken when trying to make conclusions about absolute vitamin content in human milk from Chinese mothers. Since some metabolites could not be estimated, only partial information was given on some vitamins.

## Conclusions

In conclusion, our results of B-vitamin concentrations in human milk generally agree with previously reported studies conducted in China. Our findings suggest that some regional, lactation stage, socio-economic factors including maternal BMI, regular exercise, and dietary supplement may have effects on B-vitamins in human milk from healthy Chinese mothers. In view of adequate B-vitamins concentrations in human milk as a prerequisite for healthy development in early life, further studies on accurate and complete analysis of all vitamin forms (vitamers/metabolites) are crucial for giving a more comprehensive picture of vitamins concentrations and evolution in human milk.

## Additional file

Additional file 1: **Table S1.** Multivariate linear regression models considering riboflavin and biotin concentrations in human milk after the ln transformation as the dependent variables and the other variables studied as independent variables. After adjustment for potentially confounding factors, significantly higher biotin were observed in lactating women with dietary supplement intake when compared with the other women (*p* < 0.05). Meanwhile, the concentrations of riboflavin in human milk from the lactating women with regular exercise were significantly higher than those women without (*p* < 0.05). (DOCX 16 kb)

## Abbreviations

ANOVA: Analysis of variance
BMI: Body weight index
CFC: Chinese Food Composition
CI: Confidence interval
FAD: Flavin adenine dinucleotide
FMN: Flavin mononucleotide
HPLC-MS/MS: High performance liquid chromatography-tandem mass spectrometry
IQR: Interquartile range
JFC: Japanese Food Composition
LC: Lipid chromatography
LOD: Limit of detection
LSD: Least significant difference
MING: The Maternal Infant Nutrition Growth study
MRM: Multiple reaction monitoring
MS: Mass spectrometry
PLP: Pyridoxal 5’-phosphate
QC: Quality control
Ref: Reference
SD: Standard deviation
TMP: Thiamine monophosphate
UV: Ultra violet
