# Supplementary material for: Regional, socioeconomic, and dietary factors influencing B-vitamins in human milk of urban Chinese lactating women at different lactation stages
Source: BMC Nutr. 2017 Mar 7;3:22. doi: 10.1186/s40795-017-0139-1 (PMC7050896; doi:10.1186/s40795-017-0139-1)
Supplement: Supplementary file 1 — Table S1. Multivariate linear regression models considering riboflavin and biotin concentrations in human milk after the ln transformation as the dependent variables and the other variables studied as independent variables. After adjustment for potentially confounding factors, significantly higher biotin were observed in lactating women with dietary supplement intake when compared with the other women (p < 0.05). Meanwhile, the concentrations of riboflavin in human milk from the lactating women with regular exercise were significantly higher than those women without (p < 0.05). (DOCX 16 kb) [file 40795_2017_139_MOESM1_ESM.docx]

**Table S1. Multivariate linear regression models considering riboflavin and biotin concentrations in human milk after the ln transformation as the dependent variables and the other variables studied as independent variables.**

|  | Riboflavin | |  | Biotin | |  |
| --- | --- | --- | --- | --- | --- | --- |
|  | β (95% CI) ^1^ | *P*-value |  | β (95% CI) ^1^ | *P*-value |  |
| Regular exercise |  |  |  |  |  |  |
| Yes | 0.148 (0.014, 0.281) | 0.030 |  |  |  |  |
| No | Ref |  |  |  |  |  |
| Supplement intake |  |  |  |  |  |  |
| Yes |  |  |  | 0.449 (0.206, 0.691) | < 0.001 |  |
| No |  |  |  | Ref |  |  |

CI, confidence interval; Ref, reference.

^1^ Adjusted for cities (Beijing, Suzhou, and Guangzhou cities) and lactation stages (*postpartum* 5-11 d, *postpartum* 12-30 d, *postpartum* 31-60 d, *postpartum* 61-120 d, and *postpartum* 121-240 d).

Adjusted R^2^ for riboflavin = 0.193, *p* < 0.001; adjusted R^2^ for biotin = 0.104, *p* < 0.001.
